# INDIGO – INtegrated Data Warehouse of MIcrobial GenOmes with Examples from the Red Sea Extremophiles

**DOI:** 10.1371/journal.pone.0082210

**Published:** 2013-12-06

**Authors:** Intikhab Alam, André Antunes, Allan Anthony Kamau, Wail Ba alawi, Manal Kalkatawi, Ulrich Stingl, Vladimir B. Bajic

**Affiliations:** 1 Computational Bioscience Research Center (CBRC), King Abdullah University of Science and Technology (KAUST), Thuwal, Kingdom of Saudi Arabia; 2 IBB-Institute for Biotechnology and Bioengineering, Centre of Biological Engineering, Micoteca da Universidade do Minho, University of Minho, Braga, Portugal; 3 Red Sea Research Center, King Abdullah University of Science and Technology (KAUST), Thuwal, Kingdom of Saudi Arabia; National Institute of Genomic Medicine, Mexico

## Abstract

**Background:**

The next generation sequencing technologies substantially increased the throughput of microbial genome sequencing. To functionally annotate newly sequenced microbial genomes, a variety of experimental and computational methods are used. Integration of information from different sources is a powerful approach to enhance such annotation. Functional analysis of microbial genomes, necessary for downstream experiments, crucially depends on this annotation but it is hampered by the current lack of suitable information integration and exploration systems for microbial genomes.

**Results:**

We developed a data warehouse system (INDIGO) that enables the integration of annotations for exploration and analysis of newly sequenced microbial genomes. INDIGO offers an opportunity to construct complex queries and combine annotations from multiple sources starting from genomic sequence to protein domain, gene ontology and pathway levels. This data warehouse is aimed at being populated with information from genomes of pure cultures and uncultured single cells of Red Sea bacteria and Archaea. Currently, INDIGO contains information from *Salinisphaera shabanensis*, *Haloplasma contractile*, and *Halorhabdus tiamatea* - extremophiles isolated from deep-sea anoxic brine lakes of the Red Sea. We provide examples of utilizing the system to gain new insights into specific aspects on the unique lifestyle and adaptations of these organisms to extreme environments.

**Conclusions:**

We developed a data warehouse system, INDIGO, which enables comprehensive integration of information from various resources to be used for annotation, exploration and analysis of microbial genomes. It will be regularly updated and extended with new genomes. It is aimed to serve as a resource dedicated to the Red Sea microbes. In addition, through INDIGO, we provide our Automatic Annotation of Microbial Genomes (AAMG) pipeline. The INDIGO web server is freely available at http://www.cbrc.kaust.edu.sa/indigo.

## Introduction

The Next Generation Sequencing (NGS) technologies substantially increased the throughput of genome sequencing [1-3]. Annotation of newly sequenced genomes requires a variety of experimental and computational methods [4,5], as well as integration of diverse biological information from multiple sources. Annotations stemming from information integration can be potentially used as a powerful approach in functional genomics that facilitates downstream experiments [6,7]. Data warehouses based on integrated information [8,9] are particularly useful as they open the possibility to explore content based on queries from diverse annotation attributes (e.g. genes, proteins, families, protein domains, ontologies, pathways). InterMine [10] is one of the frameworks that allows construction of such data warehouses. It has previously been applied to developing data warehouses of model genomes resulting in resources such as FlyMine, modMine, RatMine, YeastMine, etc. For more details on InterMine features and comparison to similar systems, see reference [10] and its supplementary materials.

Here, we introduce INDIGO (Integrated Data Warehouse of Microbial Genomes), a data warehouse for microbial genomes we developed, which allows integration of annotations for exploration and analysis of microbial genomes. Currently, INDIGO contains information from three species: two bacterial species, *Salinisphaera shabanensis* [11] and *Haloplasma contractile* [12], and one archaeal species, *Halorhabdus tiamatea* [13], all isolated from deep-sea anoxic brine lakes of the Red Sea. INDIGO will be regularly updated and expanded by addition of new microbial genomes from Red Sea species. 

Our contributions in this study can be summarized as follows:

- Introduction of our Automatic Annotation of Microbial Genomes (AAMG).
- Automation of data warehouse development in a high throughput manner that minimizes the intermediate steps for processing of annotation results.
- Provision to public annotations of microbial genomes being sequenced at KAUST from studies of the Red Sea environment. The number of genomes will gradually increase.

### INDIGO data warehouse

Generally, newly sequenced microbial genomes are submitted to archival databases such as GenBank [14] or EMBL [15] and later they become part of curated resources such as NCBI’s RefSeq database [16,17]. In order to help research on microbial genomes, a number of microbial data warehouses have been developed. A few examples are Integrated Microbial Genomes (IMG) [18], MicrobesOnline [19] Ensemble Genomes (www.ensemblgenomes.org) and MicroScope [20]. These publicly available data warehouses that contain microbial genomes information allow data browsing and comparison of genomes based on different sequence and functional features. On the other hand, these data warehouses are quite limited in capacity of query building and customized feature/attribute/entity list generation for more specific interrogation of information they contain.

We developed INDIGO, a data warehouse for microbial genomes using the InterMine framework Smith et al. [10] that allows extensive query building, customized feature/attribute/entity list creation and enrichment analysis for Gene Ontology (GO) concepts, protein domains and various pathways. In order to populate INDIGO with information from a newly sequenced genome, one needs a draft or complete genome assembly and functionally annotated the assembled genome. The INDIGO deployment requires the following five functions, namely, 1/ definition of a genomic data model of entities to be stored, 2/ data validation and population of the Postgres database, 3/ data integration, 4/ data post-processing, and 5/ web-application development. These five functions are synchronized through a project xml file that stores the location of different datasets, type of data sources and standard InterMine post-processing steps. 

## Results and Discussion

### Genome assembly

In our case, we reassembled previously reported [11-13], three genomes based on the NGS-generated data available from Roche and Illumina sequencers and using Roche 454 Newbler assembler (www.454.com) with scaffolding option turned on in addition to using SOAPdenovo [21] and Velvet [22]. Furthermore, we use CISA [23] to obtain consensus assemblies. We improved the resulting scaffolds using SSPACE [24], GapFiller [25] and GapCloser [21]. Applying this procedure significantly improved the assemblies by reducing the number of contigs, improved N50 parameter of all three genomes. Consequently, the redundancy in the contigs observed previously using minimus [26] is now resolved. These re-assembled contigs and associated annotations are deposited to NCBI with accession numbers AFNU00000000, AFNV00000000 and AFNT00000000 for HLPCO, SSPSH and HLRTI strains, respectively.

### Genome annotation

**Figure 1:**
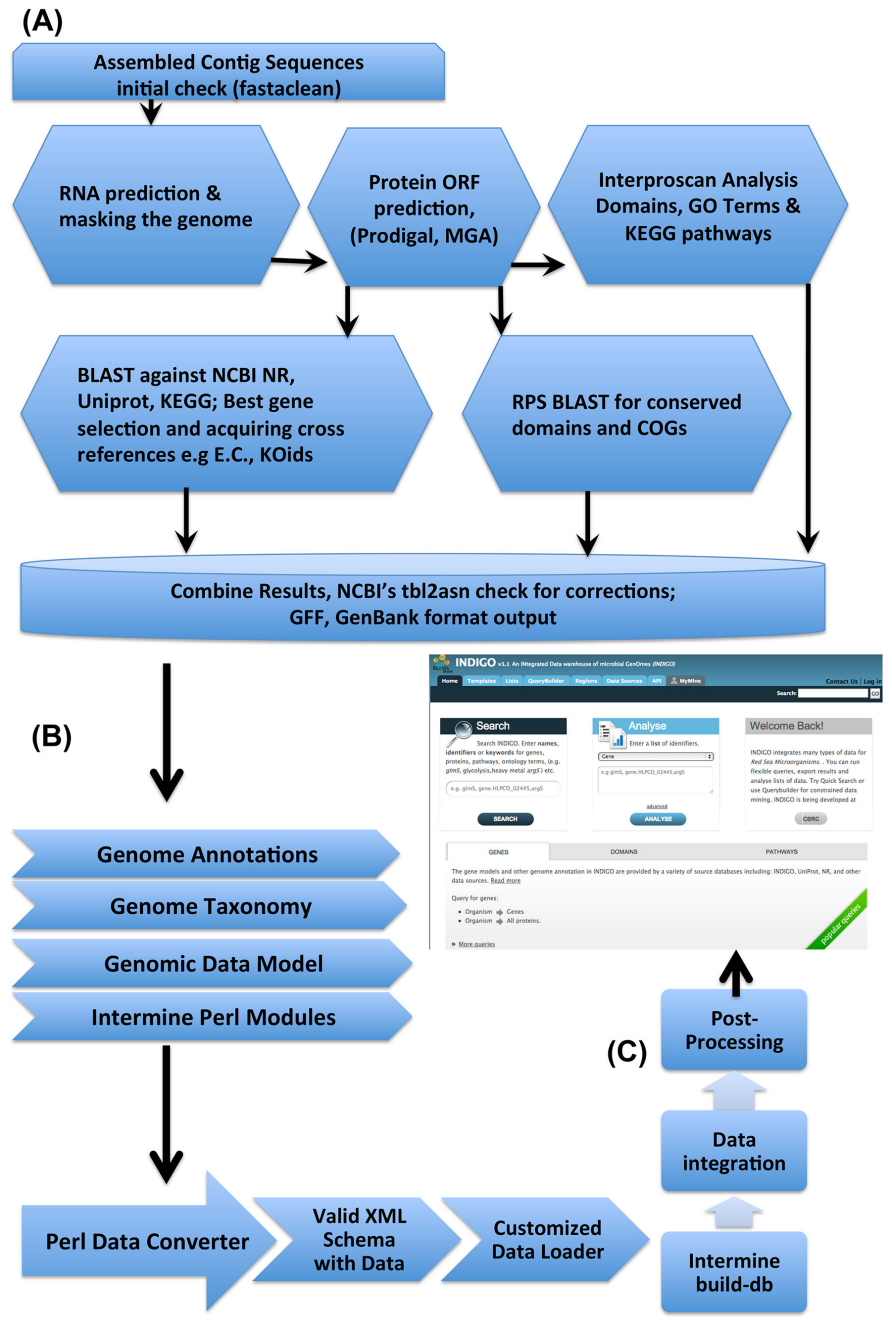
Workflow of annotation process and data warehousing. Here, the section marked (A) shows steps in the annotation process. Section (B) shows a PERL based conversion of annotations into an XML schema - validated using the class attributes and data types defined in the genomic model, and finally, section (C) shows the process of data warehouse development steps.

In our study, we performed genome annotation through a series of steps described in a workflow depicted in Figure 1. First, genomic sequences are passed through fastaclean (Exonerate package) [27]. Before the prediction of coding regions, the genome is masked for RNA using RNAmmer [28]and tRNAscanSE [29]. Predicted 16S rRNA genes are searched for in the NCBI prokaryotic 16S rRNA gene database to retrieve related taxonomic information that is later used in selecting the best BLAST hits. Open Reading Frame (ORF) prediction is performed using Prodigal [30], GeneMark [31] and MetageneAnnotator [32]. A series of BLAST [33,34] searches are then performed against the GenBank non-redundant (nr) [14], UniProt [35] and Kyoto Encyclopedia of Genes and Genomes (KEGG, [36]) databases including Reverse Position Specific (RPS) [37] searches against Conserved Domain Databases (especially COG and Prokaryotic Protein Clusters (PRK)). KEGG ortholog IDs are used to map relevant pathways and to display their presence on KEGG pathway maps. Interproscan analysis is carried out for GO terms and protein signature domains [38,39]. A check for annotation results is carried out using NCBI’s tbl2asn and errors are manually corrected. To verify origin of each contig/genomic sequence, a global scan of BLAST results of all genes is carried out and Globally Best Taxonomies (GBT) are assigned based on species from high to low ranked top hits. Ties are broken based on the higher to lower total length of alignment reported in BLAST results by each of the top scoring species.

### Benchmarking

Recently, Triplet et al. [40] thoroughly compared and benchmarked four data warehousing systems namely BioMart [41], BioXRT (mentioned in [40]), InterMine [10] and Pathway Tools [42] in a number of aspects covering accuracy, their computational requirements and development efforts. In that study, InterMine and Pathway Tools superseded other systems. InterMine obtained the highest score, where five different aspects of data retrieval for genomics research were considered, such as aggregation, algebra, graph, data integration and sequence handling. We developed INDIGO system using the InterMine framework, but we extended it by the following features not available in InterMine.

1 Development of an automatic high throughput data warehousing pipeline to process customized annotation and their validation from newly sequenced microbial genomes. As an example, we annotated and processed annotations from three extremophile genomes from Red Sea and added to INDIGO for public data mining.
2 Addition of Genome Browser functionality.
3 Addition of BLAST interface to allow comparison of external user specified sequence data to INDIGO dataset and integration of BLAST results to either explore hit genes annotations in the INDIGO data warehouse or the auxiliary genome browser.
4 We made available special hyperlinks for KEGG assigned INDIGO pathway gene sets to be shown on publication quality pathway diagrams at KEGG website.
5 and more importantly, we made available Automatic Annotation of Microbial Genomes (AAMG) pipeline for public use through the INDIGO server.

We compared INDIGO system to InterMine and few other microbial genome data warehouses such as Integrated Microbial Genomes (IMG) [18], MicrobesOnline [19] and MicroScope [20]. Table 1 shows the list of features compared as being present or not in a data warehouse. InterMine is also included in the comparison to show what are the differences between its basic framework and our INDIGO system. This comparison clearly shows the advantages of the INDIGO system complementing InterMine and providing more control to the user in integrating annotation information that is lacking in other microbial data warehouses. MicroScope microbial genome annotations data warehouse differs from INDIGO by providing a scope for manual annotation for each and every gene individually. However, it thus requires a lot of expert manpower to deal with increasing amount newly sequenced microbial genome data. MicroScope also has a number of similar features to INDIGO, but InterMine-based INDIGO system takes lead in providing several automated and powerful routes for user-defined data integration, particularly keyword, query builder or BLAST based user-controlled gene lists making, which lead to statistically robust GO, pathway or protein domain enrichment analyses.

**Table 1:** A comparison of features from different microbial data warehouses.

	INDIGO	InterMine	Integrated Microbial Genomes	Microbes Online	MicroScope
**Basic Data**					
Chromosome/Contigs	Yes	Yes	Yes	Yes	Yes
Genes	Yes	Yes	Yes	Yes	Yes
Proteins	Yes	Yes	Yes	Yes	Yes
Expression data	No	Yes	Yes	Yes	Yes
**Functional genomics**					
Gene Ontology	Yes	Yes	Yes	Yes	Yes
KEGG Pathways	Yes	Yes	Yes	Yes	Yes
Interpro Domains	Yes	Yes	Yes	Yes	Yes
Cross references	Yes	Yes	Yes	Yes	Yes
**Data Integration and Functional analysis**					
Showing assigned KEGG pathway diagrams	Yes	No	No	No	No
Individual Feature (Gene/Protein/Pathway) list generation	Yes	Yes	Yes	Yes	Yes
Multiple Feature (Gene/Protein/Pathway) list generation	Yes	Yes	No	Yes, limited	Yes
Keyword search	Yes	Yes	Yes	Yes	Yes
Keyword search against all attributes	Yes	Yes	No	Yes	No
Filter keyword search results based on categories	Yes	Yes	No	Yes	Yes
Keyword search for feature list generation	Yes	Yes	No	No	Yes
BLAST search to feature list generation	Yes	No	Yes	Yes	Yes
Query builder to user selected all/multiple feature list generation	Yes	Yes	No	No	Yes
Save / share queries	Yes	Yes	No	Yes	Yes
Feature list analysis; GO enrichment	Yes	Yes	No	No	No
Feature list analysis; Pathway enrichment	Yes	Yes	No	No	No
Feature list analysis; Protein enrichment	Yes	Yes	No	No	No
Adding additional attribute to generated lists	Yes	Yes	No	No	No
List summary functions	Yes	Yes	No	No	No
List filtering functions	Yes	Yes	Yes	Yes	Limited
List export	Yes	Yes	Yes	Yes	Yes
Save / share lists	Yes	Yes	No	Yes	Yes
Genome Browser	Yes	Yes	Yes	Yes	Yes
**Comparative Genomics**					
Compare different genomic features e.g.via keyword search	Yes	Yes	Yes	Yes	Yes
Compare sequences via BLAST	Yes	No	Yes	Yes	Yes
Compare genomes based on other tools	No	No	Yes	Yes	Yes
**Data access**					
Web server based data access	Yes	Yes	Yes	Yes	Yes
Remote access via API (PERL, JAVA, RUBY, PYTHON)	Yes	Yes	No	Yes	No
Bulk Download	Yes	Yes	Yes	Yes	Yes
User selected single feature list based download	Yes	Yes	Yes	Yes	Yes
User integrated feature list based download	Yes	Yes	No	No	Yes, limited.
**Genome Annotation**					
Public microbial genome annotation	Yes	No	Yes	Limited, uses rast and takes six months	Annotation_editor (manual)
User genome annotation job history	Yes	No	Yes	Yes	Manual
**Genome Annotation features**					
operon finding	No	No	Yes	Yes	Yes
promoter/terminator finding	No	No	Yes	No	Yes
RNA detection (rRNA/tRNA)	Yes	No	Yes	No	Yes
Protein gene prediction (multiple methods)	Yes	No	Yes	No	Yes
RNA vs. Protein overlap resolution	Yes	No	Yes	No	Yes
HPC BLAST for Proteins to UniProt	Yes	No	No	Yes	Yes
HPC BLAST for Proteins to NCBI NR	Yes	No	No	Yes	No
HPC BLAST for Proteins to NCBI COG	Yes	No	Yes	Yes	Yes
HPC BLAST for Proteins to NCBI CDD	Yes	No	No	No	No
HPC BLAST for Proteins to KEGG	Yes	No	Yes	Yes	Yes
HPC Interproscan domain finding for Proteins	Yes	No	Yes	Yes	Yes
Global Best Taxonomy (GBT) distribution analysis	Yes	No	No	No	No
Annotation data integration to GFF format	Yes	No	Yes	No	No
Annotation data integration to GenBank format	Yes	No	No	Yes	Yes
Annotation data integration to TBL format	Yes	No	No	No	Yes
Annotation data checking using tbl2asn	Yes	No	No	No	No
Annotation data process to NCBI sqn submission format	Yes	No	No	No	No
Annotation data packing into validated xml for data warehouse	Yes	No	No	No	No
Hierarchical classification of COG annotations and visualization	Yes	No	No	Yes	No
Hierarchical classification of GO annotations and visualization	Yes	No	No	No	No
Hierarchical classification of GBT annotations and visualization	Yes	No	No	No	No
Hierarchical classification of InterPro domains annotations and visualization	Yes	No	No	Yes	Yes
Hierarchical classification of ALL annotations and visualization	Yes	No	No	No	No
Immediate access to all data files and visualizations	Yes	No	No, sso accounts	Yes	Yes

### Benchmarking genomic annotations

To assess the quality and volume of annotations produced using our AAMG pipeline, we compare AAMG annotation results based on three publicly available datasets. Two of these datasets, namely *Escherichia coli* (*E. coli*) K12 strain and *E. coli* TY2482 strain, were recently considered in benchmarking two different annotation pipelines [43]. The third dataset is a very small genome, *Candidatus* Carsonella *ruddii* DC[44].

**Figure 2:**
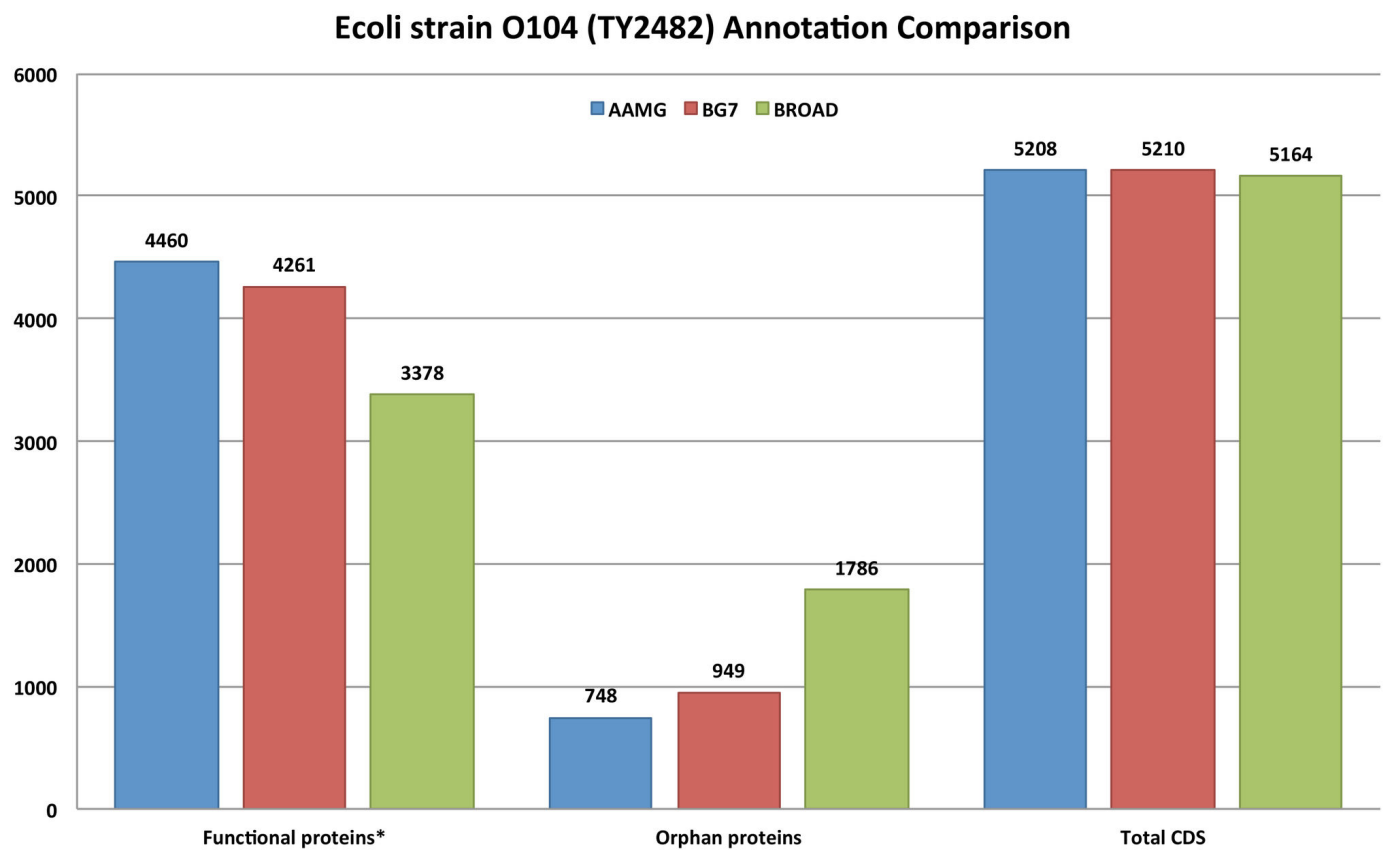
Annotation comparison for *E. coli* O104 (TY2482) among AAMG pipeline, BG7 and reference annotation set from Broad Institute. Regarding the CDS annotation AAMG ranks second (with only 2 CDS region less annotated than BG7), while in annotation of orphan (hypothetical) CDS products (the less the better) and in annotation of functional (non-hypothetical) CDS products (the more the better) AAMG performs the best.

Recent outbreak of *E. coli* in Germany triggered the sequencing of *E. coli* O104 [45], the cause of enterohemorrhagic diarrhea. Sequencing was carried out in BGI (the strain TY2482) and multiple groups annotated this sequence. The annotation produced by AAMG pipeline for *E. coli* TY2482 is compared with annotations available from BG7 [43]. BG7 pipeline compared the annotations considering an annotations set available from Broad Institute website, http://www.broadinstitute.org/annotation/genome/Ecoli_O104_H4/assets/Ecoli_TY_2482_BGI.gbk. Results depicted in Figure 2, show that we achieve comparable performance in gene calls. Furthermore, considering the annotation in assigning gene product names, our annotation shows a significant increase in non-hypothetical products as compared to Broad Institute annotation and BG7. 

BG7 compared its *E. coli* O104 (TY2482) annotation results to RAST-based annotations considering Broad Institute annotation as a gold standard. It was reported for *E. coli* TY2482 assembly version 4 [43] that BG7 predicted 5210 CDS genes, 163 false negatives and 271 false positives, while the number of genes obtained with RAST was 5446 with 116 false negatives and 321 false positives. We report AAMG-based annotation of *E. coli* TY2482 (see Supplementary materials) showing about the same number of genes predicted as BG7, but with higher numbers of functional (non-hypothetical) products and smaller number of orphan (hypothetical) genes when compared to the Broad Institute reference annotations. 

In addition to *E. coli* O104 (TY2482), we also compared our results in comparison to existing annotations in NCBI for E. coli K12 and another much smaller genome, *Candidatus* Carsonella *ruddii* DC. Results show that our annotation pipeline is able to minimize hypothetical genes names through scanning multiple full protein and domain databases. Our gene calls are also very close to the existing annotations. Table 2 shows this annotation comparison.

**Table 2:** Results of AAMG Annotations compared with NCBI or BROAD institute sets.

	*E. coli* K12 W3110		*E. coli* TY2482		*C. ruddii* DC	
Gene calls	AAMG	NCBI	AAMG	BROAD	AAMG	NCBI
CDS	4340	4337	5208	5164	190	207
rRNA	22	22	22	22	3	3
tRNA	82	86	97	97	27	28
Total	4444	4445	5327	5288	220	238
False Negatives	235	236	50	11	4	22
Functional genes	3866	3730	4591	3502	182	191
Orphan genes	578	715	736	1786	38	47
**Gene calls**	**Genes by AAMG**	**% of NCBI genes**	**Genes by AAMG**	**% of BROAD genes**	**Genes by AAMG**	**% of NCBI genes**
Detected* identical	3876	87.20	5172	97.81%	205	86.13
Detected similar**	333	7.49	105	1.99%	11	4.62
Not Detected	236	5.31	11	0.21%	22	9.24
Total	4445		5288		238	

^*^ Genes are identical when both start and stop positions are exactly the same.

^**^ Genes are similar if start or stop positions are in the same region with an offset up to 50 bases.

Our annotations for these three genomes are available as a material at http://www.cbrc.kaust.edu .sa/indigo_data/. Data files and results are visualized using interactive graphs based on modified Krona package [46].

## Methods and Analysis

### Genomic data model

InterMine provides a core genomic data model defined with several genome entities, their attributes, syntax and relationships. We extend this core genomic model to fit our needs so as to cater to all types of annotations we receive from our annotation process. This includes data types and relationships between entities to be stored, such as attributes for organism, contigs, genes, CDS, protein domains, pathways and cross references. An example of genomic data model is provided in the materials at the website.

### Data validation and population of the Postgres database

InterMine provides a built-in setup for complex data integration, post-processing and web-application development. Data integration is heavily dependent on genomic model defined with data types and relationships between entities to be stored. Once a genomic model is defined, one can perform a check for the annotation that is to be loaded into the database. Our system first validates the annotation in reference to the defined genomic model using InterMine’s Model and Document Perl Modules. It then prepares an xml schema filled with data that is ready to be loaded to the backend Postgres database. InterMine loads data into the database using pre-defined ‘sources’ for different types of data packed in different formats. For example, to load genes data packed in the gff format, a Java-based data converter is available, but it assumes specific tags and fields. For customized data loading we developed prokaryotic-annots-xml, available as Supplementary material here, which allows loading of our validated annotations packed in xml format. InterMine’s build-db setup reads the generated annotation using prokaryotic-annots-xml source and loads the data by defining and populating different annotation tables automatically. 

### Data integration, post-processing and web-application development

Data integration in the InterMine’s framework is a crucial step. It integrates data from sources provided in the project xml file and performs multiple checks (e.g. the absence of empty fields, the absence of duplicate data being stored, etc.). We only provide database identifiers in the annotation xml, for example, for GO or Interpro protein domains (IprD), and InterMine system integrates corresponding detailed annotations from complete GO or IprD source files defined in the project xml. 

There are several built-in post-processing steps available in the InterMine framework such as create-search-index, transfer-sequences, etc., that allow for quick indexing of the data. For INDIGO, in order to have all the functionality available, we run all post-processing steps. Table 3 shows different stages in our data warehouse development along with the processing time using InterMine framework.

**Table 3:** Data warehouse development stages using InterMine.

**INDIGO Steps**	**Action**	**Time (seconds)**
build database tables	build-db	2
data integration	prokredsea-HLPCO-largexml	59
data integration	prokredsea-HLRTI-largexml	61
data integration	prokredsea-SSPSH-largexml	68
data integration	Sequence ontology	56
data integration	interpro	164
data integration	Gene ontology	1043
post-processing	create-references	28
post-processing	make-spanning-locations	21
post-processing	create-chromosome-locations-and-lengths	40
post-processing	transfer-sequences	89
post-processing	create-bioseg-location-index	15
post-processing	create-attribute-indexes	38
post-processing	summarise-objectstore	31
post-processing	create-autocomplete-index	20
post-processing	create-search-index	59
total time taken		1794

Web-application templates are available in the InterMine framework and we customized them to fit our requirements. For example, report pages for genome features such as genes, proteins, domains, and pathways are customized according to the data available including hyperlinks to external databases. One of the interesting external links allows for displaying KEGG pathway diagrams showing presence of the KEGG Ortholog ids to which the explored genome is mapped. Such a display shows which elements of the reference pathways are present or missing from the genome being examined. InterMine allows packaging of the web-application as a Web Application Archive or WAR-file that is then deployed on the Tomcat Apache server (http://tomcat.apache.org). 

### INDIGO Web Interface Organization

INDIGO is equipped with a number of features that allow for the exploration and analysis of the deposited information. INDIGO front-end is organized into different main pages accessible through tabs, namely ‘Home’, ‘Templates’, ‘Lists’, ‘QueryBuilder’, ‘Regions’, ‘Data’, ‘API’, ‘BLAST’ and ‘MyMine’, where each tab provides access to the data in different ways. 

The INDIGO ‘Home’ page presents options for quick keyword search, analysis of a list of genes/proteins and the use of predefined templates to perform queries. The ‘Template’ tab shows all predefined templates to perform queries such as Organism->Protein which help to obtain all proteins in a genome. The ‘Lists’ tab provides access of all feature types; for example, selecting a feature type such as gene, protein, protein domain, etc. and providing a list of identifiers, makes a list of items with default attributes that can be saved or further analyzed. The ‘QueryBuilder’ tab provides the most exhaustive functionality for building queries in INDIGO and it provides more control to include (show option) or limit (constrain option) for different feature types and their attributes. ‘Regions’ tab provides access to all features present in a given genomic coordinate range. ‘Data’ tab provides general information about the genomic data sets included in the data warehouse, e.g. genome assembly statistics, counts and links to contigs, ORF sequences, archaeal/bacterial genome completeness statistics based on counts of archaeal/bacterial core COGs [47], minpath-based [32] KEGG pathway association statistics, etc. API provides details on how to access data warehouse using PERL, Python, Ruby and Java programming languages. The ‘BLAST’ tab allows users to carry out Basic Local Alignment Search Tool (BLAST) based similarity search for a DNA or protein sequence of interest with genes in INDIGO. The result of BLAST search is shown as a list where users can save and select an individual or all hits for further GO, Pathways or protein domain enrichment analysis. Finally, ‘MyMine’ shows an interface to Automatic Annotation of Microbial Genomes (AAMG) pipeline, user-specific lists and queries performed and saved by a user once the user creates an account on the system. Individual report pages for genomic features provide details and hyperlinks for several related attributes including JBrowse [48] visualization. 

### Use of INDIGO and its features

When analyzing a new genome, majority of questions can be summarized in ‘What’, ‘Where’ and ‘How’ context. For example, to see whether a gene, protein, protein domain, GO term or a pathway of interest can be found in INDIGO, a search mechanism can help. For ‘Where’ context questions, the ‘Region’ search option in INDIGO can list all the genomic features in a given range of genomic coordinates. For complex questions of the type e.g. ‘What is a list of genes involved in pathway X and what are their protein domains and associated GO terms’ more control on what is being searched is needed and here it is provided through Query Builder. More details on features of INDIGO, such as a quick and easy keyword search, query builder search, analysis of genomic feature (such as gene, protein, protein domains) lists, genomic region search, and enrichment analysis for GO, protein domains and pathways, are shown in few examples in what follows.

### Keyword and Query Builder Search

**Figure 3:**
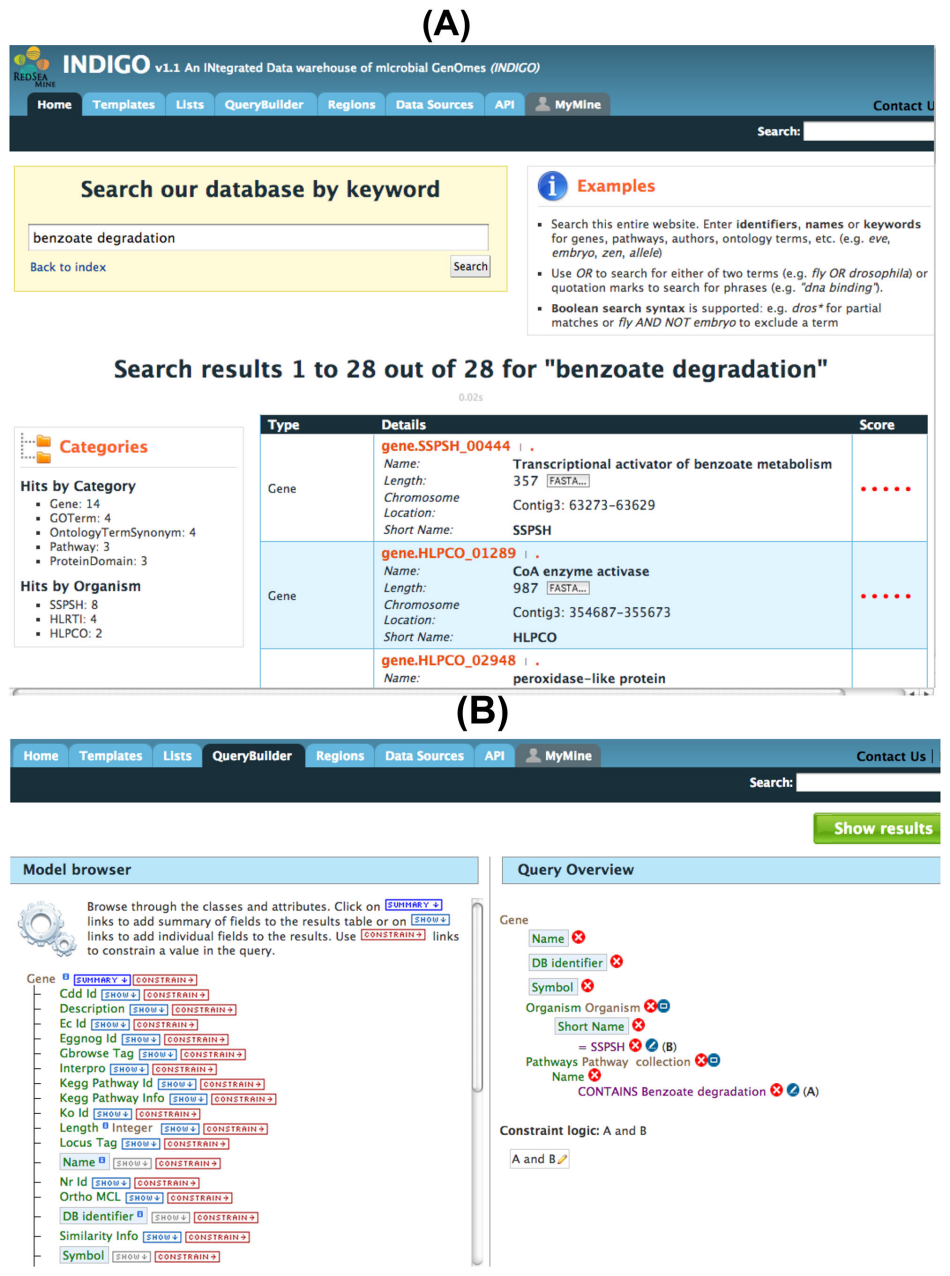
A) Keyword and B) Query builder search interface to INDIGO. The keyword search interface shows an example of the search for “benzoate degradation”. Results are categorized on the left side of the resulting page, showing the number of hits found for genes, domains, pathways, etc. These results are further categorized into hits per genome for different organisms. Clicking on any of these categories shows filtered results. The query builder interface has an option to include or constrains an annotation class attribute, e.g. pathway name is constrained for “benzoate degradation”, while the organism attribute ‘short name’ is constrained to “SSPSH”. The annotation feature class attributes to be included in the result list here are gene db identifier, symbol, organism’s short name and pathway name. User can select any of the available annotation class attributes making it possible to integrate annotation from several different sources. Results of constrained query builder search are shown as a list. There are summary and filter options on the list page that allow a user to further analyze these results.

In the INDIGO system, a keyword search, as well as a more extensive query builder search option, are provided. The keyword search option provides a very simple interface to the underlying annotation data. It is very fast since all the keywords in the database are indexed. Query builder, however, provides more control over annotation classes and attributes to be searched, constrained or viewed. It is possible to combine several queries through constraint logic. Figure 3 shows an example of Query-Builder interface to INDIGO.

### Region Search

**Figure 4:**
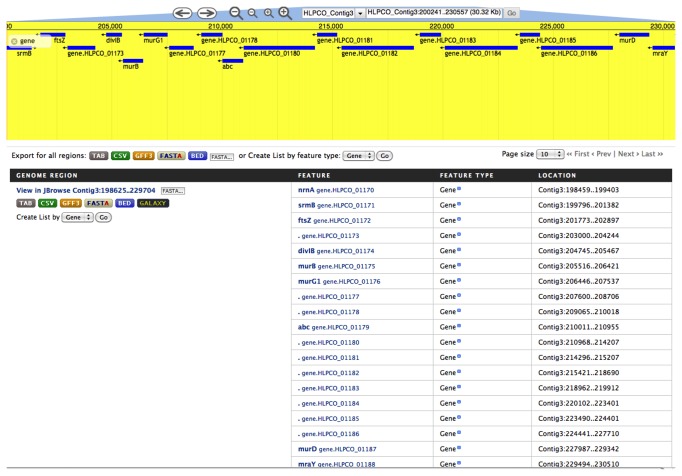
Region search interface. This figure shows features (genes) for a region using coordinates (Contig3:198625-229704) from organism *Haloplasma* *contractile* (HLPCO). This region shows the cell Division and Cell Wall (DCW) biosynthesis gene cluster. An integrated genome browser view available via Region search results page, shows here the arrangement of genes in this region of the contig from HLPCO . The table below this section shows genome region, data export options, basic details of the feature (genes), type of features and their location on the genome. The create list by feature link saves this gene list in the data warehouse for further analysis. This list stays permanently if the user is logged in.

In order to find out characteristics of a particular genomic region, one can use region search. When coordinates for the specific genomic region are provided, the region search allows for selection of additional upstream and downstream regions, as well as features like gene or intergenic region, etc. (Figure 4). Results can be exported in several different formats. We integrated JBrowser [48] based visualization of our genomic features in the region search results page. In the genome browser users can look up gene names or particular coordinates of genomes to view underlying features. Available tracks are DNA, gene and InterPro domains.

### Analysis of Lists

**Figure 5:**
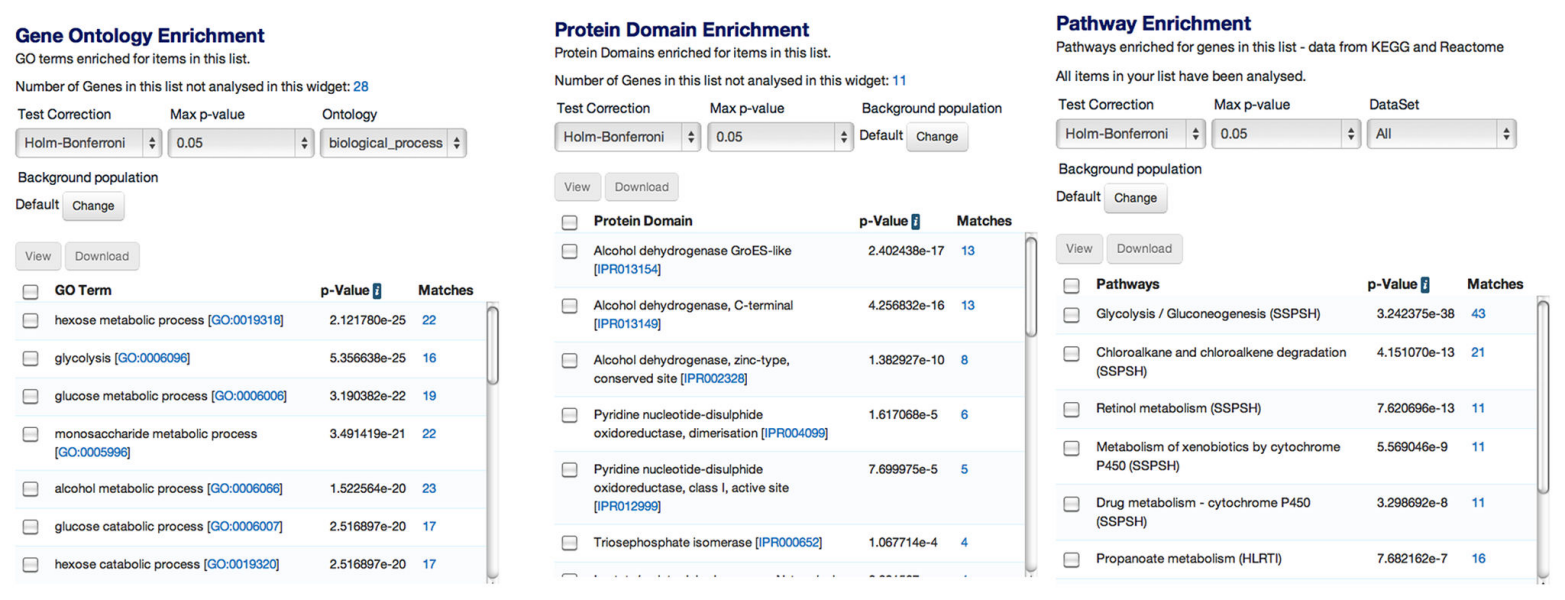
A) Gene Ontology, B) Protein Domain and C) Pathway enrichment analysis. The figure shows a snapshot obtained in case when a term “cell cycle” was searched through the keyword search option and resulting genes were saved in a list that shows enrichment of GO, protein domain and pathways in comparison to the rest of the data in INDIGO. The number of hits shown for reach category can be saved as lists for further analysis.

INDIGO makes use of different types of lists. For example, a list could be the list of genes/proteins, or protein domains, etc. Results from keyword search or query builder, can be saved as a list. A click on the saved list link automatically shows GO, protein domain and pathway enrichment, as shown in an example in Figure 5.

The user is also able to save all enriched genes, make sub-lists, view individual gene report pages, or export results. Enrichment analysis provided for a list includes p-values based on hypergeometric distribution with several multiple testing correction options (for further details on the enrichment process, see https://intermine.readthedocs.org/en/1.1/embedding/list-widgets/enrichment-widgets/). 

### Current content of INDIGO

The King Abdullah University of Science and Technology (KAUST) has in its focus areas the biodiversity and microorganisms of the Red Sea. INDIGO is populated with information from three extremophiles from the Red Sea, whose genomes have been previously reported by our team [11-13]. The details are provided in what follows.

### Red Sea environment

The Red Sea is one of the warmest, most saline and most nutrient-poor oceanic water bodies in the world [49,50]. It also hosts several deep-sea anoxic brine lakes, which are considered some of the most remote and extreme environments on Earth [51]. The brines markedly differ from overlying seawater and are unique due to the combination of multiple extremes namely high salinity (7-fold increase), high temperature (up to 70°C), high concentration of heavy metals (1,000- to 10,000-fold increase in concentration), high hydrostatic pressure and anoxic conditions. Despite this combination of multiple environmental extremes, they have been shown to harbor a very high biodiversity, with identification of several new phylogenetic lineages and isolation of several new extremophiles [51]. 

### Three Red Sea extremophiles in INDIGO

Three extremophilic microbes, previously isolated from the deep-sea anoxic brine lakes, were selected as part of a genome-sequencing project due to their phylogenetic position, peculiar features and unique biotope. Analysis of their draft genomes provides us with a first glimpse on some of their unusual characteristics and the ways they cope with living in such a harsh environment [11-13]. 

### *Salinisphaera shabanensis*

*Salinisphaera shabanensis* was isolated from the brine-seawater interface of Shaban Deep [52]. It represented a new order within the *Gammaproteobacteria*, and displayed a remarkable physiological versatility. Indeed, *Salinisphaera shabanensis* had quite broad growth ranges for oxygen, temperature, NaCl, pressure, and, to a smaller degree, pH [52].

### *Haloplasma contractile*

*Haloplasma contractile* was isolated from the brine-sediment interface of Shaban Deep. Phylogenetically it represented a novel lineage within the Bacteria with branching position between the Firmicutes and Tenericutes (Mollicutes), with no close relatives [53]. The most striking feature of *Haloplasma* is its unusual morphology and unique cellular contractility cycle.

### *Halorhabdus timatea*

*Halorhabdus tiamatea* was isolated from the brine-sediment interface of the Shaban Deep [54] using fluorescence *in situ* hybridization coupled with the “optical tweezers” technique [55,56]. It was described as a new species and is currently still the only member of the Archaea to have been described from a deep-sea anoxic brine.

### Features of the three Red Sea extremophiles from INDIGO

In Table 4 we present summary of the basic genomic features associated with the re-assembly of three microorganisms included in INDIGO. 

**Table 4:** Basic annotated features of the three Red Sea extremophiles in INDIGO.

**Organism**	**Contigs**	**N50**	**ORFs**	**rRNAs**	**tRNAs**
*Haloplasma contractile*	34	347868	3036	4	27
*Halorhabdus tiamatea*	72	58136	3287	3	40
*Salinisphaera shabanens*	41	129079	3530	3	46

INDIGO provides easy and quick access to genomic annotations of microbial species at the levels of chromosomes, genes, and proteins, as well as to the associated GO and pathways. The top 10 pathways based on the number of genes assigned to each of these extremophiles as found by INDIGO are shown in Table 5.

**Table 5:** Top 10 pathways from each of the three extremophiles.

***Haloplasma contractile***	Genes	***Salinisphaera shabanens***	Genes	***Salinisphaera shabanens***	Genes
ABC transporters	115	Two-component system	182	ABC transporters	88
Purine metabolism	69	ABC transporters	131	Purine metabolism	74
Two-component system	67	Purine metabolism	96	Ribosome	64
Pyrimidine metabolism	56	Methane metabolism	78	Pyrimidine metabolism	60
Ribosome	56	Oxidative phosphorylation	75	Oxidative phosphorylation	55
Tyrosine metabolism	52	Butanoate metabolism	73	Amino sugar and nucleotide sugar metabolism	53
Amino sugar and nucleotide sugar metabolism	50	Benzoate degradation	71	Two-component system	50
Starch and sucrose metabolism	49	Fatty acid metabolism	70	Methane metabolism	46
Methane metabolism	46	Arginine and proline metabolism	63	Starch and sucrose metabolism	40
Histidine metabolism	40	Pyruvate metabolism	60	Cysteine and methionine metabolism	39

### Examples of exploration of Red Sea Extremophiles via INDIGO

#### Region search: Analysis of the *dcw* gene cluster in *Haloplasma contractile*

The most remarkable features of *Haloplasma contractile* include its unusual morphology and contraction cycle and these provided clear targets for genomic-based exploration. While some aspects of the genetic control of cellular morphology remain unclear, the *dcw* gene cluster seems to play a central role. Gene context is particularly relevant, as morphology is impacted by presence or absence of specific genes, together with relative position and distance within this gene cluster [57,58]. Using the region search of INDIGO we were able to locate the *murD* - one of the central genes of the *dcw* gene cluster. Furthermore, we analyzed the genomic context of *murD* (upstream and downstream regions) and successfully demonstrated multiple gene insertions and disruption of the *murD-ftsW-murG* gene order, see Figure 4. Such a disruption would justify the atypical morphology of *H. contractile* as they have been previously implicated in all non-rod morphologies currently known [58,59].

#### Pathway search: Benzoate degradation in *Salinisphaera shabanensis*

Aromatic compounds are abundant, widely distributed and known to constitute some of the most prevalent and persistent pollutants in the environment [60]. Some microbes have evolved complex machinery and metabolic pathways for their degradation [61] with benzoate being widely used as a model compound for studying their catabolism. 

**Figure 6:**
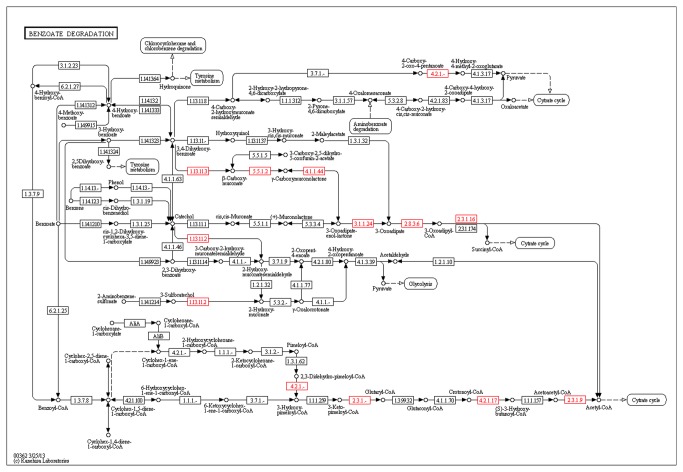
Benzoate degradation in *Salinisphaera* *shabanensis*. The genes from *Salinisphaera* *shabanesis* associated with Benzoate degradation pathway by INDIGO are shown in Red. INDIGO developed a functionality, available for all pathways present in INDIGO, that generates a specific URL to automatically display KEGG Orthologs from INDIGO on to pathway diagrams at KEGG webserver.

Based on previous detection of a variety of complex hydrocarbons in Shaban Deep [62], we looked into genomic-based evidences for possible aromatic compound catabolic capability.  The use of the query builder search (Figure 3) in INDIGO and its mapping onto KEGG pathway (Figure 6) led us to promising results, with the identification of an almost complete branch of the benzoate degradation pathway in *Salinisphaera shabanensis*. Such valuable information obtained through the simple use of INDIGO will aid in search for target missing genes and/or design downstream laboratorial experiments to confirm the functionality of this pathway, and explore possible future applications. 

## Conclusion

The new data warehouse system, INDIGO, enables users to combine information from different sources of annotation for further specific or general analysis. This data warehouse of Red Sea microorganisms currently contains information about three genomes (two bacterial and one archaeal). Considering the unique biodiversity present in the Red Sea, KAUST has undertaken a large sequencing effort starting from metagenomes to cultured and uncultured single cell amplified genomes. The plethora of sequencing data produced requires a high throughput assembly, annotation and data warehousing pipelines. This work shows the basic framework through which these pipelines can be used in a high throughput manner to properly warehouse the increasing amount of data for targeted studies. Additional genomes will include both, genomes of pelagic bacteria and archaea, as well as more extremophiles from the brine pools of the Red Sea.
